# Bioaugmentation has temporary effect on anaerobic pesticide biodegradation in simulated groundwater systems

**DOI:** 10.1007/s10532-023-10039-0

**Published:** 2023-07-13

**Authors:** Andrea Aldas-Vargas, Jannigje G. Kers, Hauke Smidt, Huub H. M. Rijnaarts, Nora B. Sutton

**Affiliations:** 1 Environmental Technology, Wageningen University & Research, P.O. Box 17, 6700 EV Wageningen, The Netherlands; 2 Laboratory of Microbiology, Wageningen University & Research, P.O. Box 8033, 6700 EH Wageningen, The Netherlands

**Keywords:** 2,4-D, Biodegradation, Bioaugmentation, Pesticides, Groundwater

## Abstract

Groundwater is the most important source for drinking water in The Netherlands. Groundwater quality is threatened by the presence of pesticides, and biodegradation is a natural process that can contribute to pesticide removal. Groundwater conditions are oligotrophic and thus biodegradation can be limited by the presence and development of microbial communities capable of biodegrading pesticides. For that reason, bioremediation technologies such as bioaugmentation (BA) can help to enhance pesticide biodegradation. We studied the effect of BA using enriched mixed inocula in two column bioreactors that simulate groundwater systems at naturally occurring redox conditions (iron and sulfate-reducing conditions). Columns were operated for around 800 days, and two BA inoculations (BA1 and BA2) were conducted in each column. Inocula were enriched from different wastewater treatment plants (WWTPs) under different redox-conditions. We observed a temporary effect of BA1, reaching 100% removal efficiency of the pesticide 2,4-D after 100 days in both columns. In the iron-reducing column, 2,4-D removal was in general higher than under sulfate-reducing conditions demonstrating the influence of redox conditions on overall biodegradation. We observed a temporary shift in microbial communities after BA1 that is relatable to the increase in 2,4-D removal efficiency. After BA2 under sulfate-reducing conditions, 2,4-D removal efficiency decreased, but no change in the column microbial communities was observed. The present study demonstrates that BA with a mixed inoculum can be a valuable technique for improving biodegradation in anoxic groundwater systems at different redox-conditions.

**Graphical abstract:**

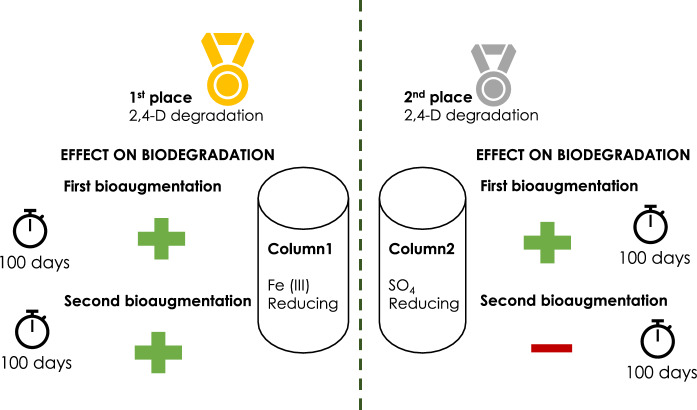

**Supplementary Information:**

The online version contains supplementary material available at 10.1007/s10532-023-10039-0.

## Introduction

Groundwater is the main source for the production of drinking water around the world. In Europe, there are regulations to secure safe drinking water production from clean sources (Drinking Water Directive 98/83/EC) (European Comission 2006), pesticides nonetheless percolate into groundwater systems (Heuvelink et al. 2010; Van Eerdt et al. 2014). Pesticide application varies in dosage rate, time and space, resulting in heterogeneous pesticide distribution (Schipper et al. 2008). This heterogeneity in pesticide use, has also been observed in the presence of pesticides at micropollutant concentrations in European groundwater (Loos et al. 2010). Specifically in groundwater in The Netherlands, the more frequently detected compounds for already several years are 2,6-dichloro-benzamide (BAM), bentazone and mecoprop-p (MCPP) (Sjerps et al. 2019; Swartjes and Van der Aa 2020).

Biodegradation is a sustainable approach to remove pesticides at micropollutant concentrations from contaminated aquifers (Meckenstock et al. 2015). However, active biodegradation of pesticides in groundwater is limited due to the presence of anoxic conditions, low nutrient concentrations and low quality and concentration of carbon substrates (Fenner et al. 2013; Helbling 2015). That, in combination with the heterogenous distribution of pesticides in the system (Schipper et al. 2008; Sjerps et al. 2019), impede the natural development of a stable pesticide degrading microbial community in the field (Vandermaesen et al. 2016). Literature studies demonstrated that exposure is a determinant factor for the ability of microbial communities to biodegrade contaminants (Lipthay et al. 2003; Poursat et al. 2020; Tuxen et al. 2002). Taking into account the above mentioned limitations, groundwater may require the use of amendments to support the biodegradation of pesticides.

The role of environmental conditions in the development of a degrading microbial community was previously studied demonstrating that the naturally occurring redox conditions in the field are the main selective pressure for field microbial communities (Aldas-Vargas et al. 2022a). For that reason, the influence of different electron acceptors in combination with a labile carbon source was studied as a biostimulation strategy in mesocosms simulating groundwater systems (Aldas-Vargas et al. 2021). Results demonstrated the importance of having a carbon source as well as a suitable electron acceptor. However, results also showed that natural groundwater microbial communities are unable to biodegrade micropollutants, even with amendments. Thus, it was evident that biostimulation needed to be complemented with other remediation strategies to improve the pesticides biodegradation in groundwater systems. Therefore, based on previous research, complementary remediation strategies should consider different groundwater redox conditions and also the addition of a labile carbon source.

Bioaugmentation (BA) is a bioremediation technique that increases the pesticide-degrading activity by the addition of microorganisms with a desired catabolic activity (Cycoń et al. 2017). So far, BA has been validated for the individual biodegradation of some pesticide-metabolites such as BAM (Ellegaard-Jensen et al. 2020), and also other pesticides such as 2,4-dichlorophenoxyacetic acid (2,4-D) (Dai et al. 2015). However, many existing studies perform BA for individual compounds under oxic conditions, in either sand filter columns or soil microcosms (Albers et al. 2018; Ellegaard-Jensen et al. 2020; Horemans et al. 2017; Schultz-Jensen et al. 2016). Conducting BA experiments in groundwater systems which are mainly anoxic environments, with depth-dependent redox conditions is therefore innovative and relevant for the biodegradation of pesticides in natural aquifers. Since groundwater aquifers tend to be oligotrophic environments, they tend to possess less carbon and nutrients compared to sand filters limiting thus the overall microbial activity (Griebler and Lueders 2009). Therefore, it is necessary to investigate how BA can be applied in relevant field conditions for the successful remediation of groundwater contaminated with micropollutants.

Central to effective BA is maintaining pesticide biodegradation activity, which is challenging in open, oligotrophic and anoxic groundwater systems. For instance, autochthonous and inoculated microorganisms compete for nutrients and substrates; this competition determines if the added inoculum survives or if biodegradation activity is retained (Bouchez et al. 2000; Cycoń et al. 2017; Wenderoth et al. 2003). The aforementioned BA studies where single strains were used for BAM and 2,4-D degradation, showed that in one case, the added strain disappeared in the system (Dai et al. 2015), and in the other case, the strain lost biodegradation activity (Ellegaard-Jensen et al. 2020). These results highlight the importance of further experimentation using BA with a mixed inoculum instead of a single strain for treating micropollutants with bioremediation. The addition of a mixed inoculum for the BA of anoxic and oligotrophic groundwater systems, has to the best of our knowledge not been conducted yet.

In this study, we investigated pesticide biodegradation at low concentrations under anoxic conditions. We performed BA with a mixed inoculum on two column bioreactors that simulate groundwater systems at different redox conditions. We evaluated the effects of BA in terms of removal efficiency of one pesticide metabolite and two pesticides: BAM, MCPP and 2,4-D. These compounds have been reported to threaten Dutch drinking water sources (Swartjes et al. 2016) and are usually found together, as simulated in the current experiment. Microbial community dynamics were studied before and after BA to understand survival of the inocula within the existing microbial communities as well as to identify the presence of known-degraders during the experiment. The results of this work provide insights into the potential use of BA using a mixed inoculum for remediating pesticide-polluted anoxic groundwater under two naturally occurring redox conditions.

## Materials and methods

### Chemicals and reagents

The pesticide metabolite BAM and the pesticides MCPP and 2,4-D, were purchased from Sigma-Aldrich (USA). Green compost (Van Iersel Compost, The Netherlands), with a composition of 50% screened wood, 25% grass litter, and 25% leaf litter was used for dissolved-organic-carbon (DOC) extraction as described in Luo et al. (2019).

### Experimental set-up

#### Simulated groundwater systems

**Fig. 1:**
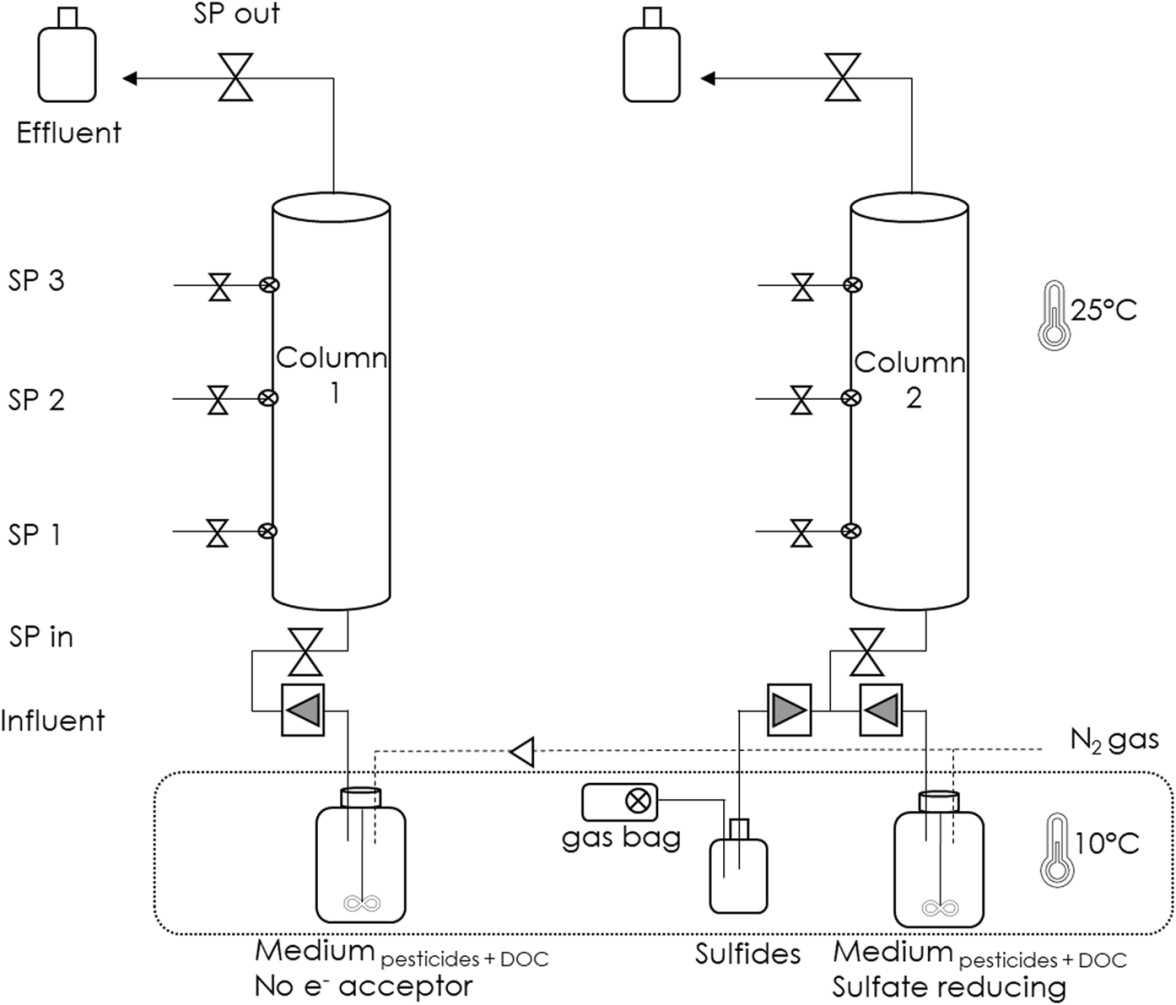
Column experimental setup simulating groundwater systems with different redox conditions. Column1 received no external electron acceptor amendment and column2 was sulfate-reducing (SO_4_^−^ at 1190 mg/L concentration). The concentration of each pesticide (BAM, 2,4-D and MCPP) was 1 mg/L. The concentration of DOC was 10 mgC/L. The column experiments lasted for more than 800 days.* SP* sampling point

Two continuously-fed, up-flow column reactors filled with aquifer material under different anoxic redox conditions were used to simulate groundwater systems (Fig. 1). The glass columns were 10 cm internal diameter and 50 cm long. Both columns were placed in a cabinet with a temperature of 25 °C covered with light-blocking Plexiglas to prevent photodegradation. Medium was refreshed twice per month. The medium was prepared according to a previous study (Luo et al. 2019) and either no additional electron acceptor (column1) or sulfate (column2) was supplemented. In the case of no additional electron acceptor, the naturally-present iron from the aquifer material most likely served as electron acceptor. The concentration of each pollutant (BAM, 2,4-D and MCPP) in the medium was 1 mg/L, and the DOC concentration was 10 mg C/L. The medium bottles were placed in a fridge at 10 °C to prevent microbial activity outside the columns. Also, medium bottles were continuously stirred and purged with N_2_ gas to keep the medium anoxic. The medium flow rate was set at 8 mL/h, and the hydraulic-retention-time (HRT) was 6.87 days for column1 and 7.70 days for column2, due to differences in the sediment content between the columns. The experimental pH of columns 1 and 2 was 7.8 and 8.2, respectively. Thus, 2,4-D (pKa = 3.4) and MCPP (pKa = 3.1) were in ionized form at the experimental pH. The simulated groundwater systems were operated for 801 days for column1 and 822 days for column2. The columns were used to determine the natural pesticide biodegradation activity of the system under the different redox conditions before and after BA.

#### Bioaugmentation in simulated groundwater systems

##### Batch enrichments

For each BA inoculation, samples from diverse WWTPs were used to create pesticide degrading mixed enrichments assuming that a diverse inocula possess diverse metabolic capacity. The three pesticides (BAM, 2,4-D and MCPP) were used in a mixture since a degrading inoculum for different pesticides present in the field was desired. The enrichment process was done in multiple microcosms per redox condition with the same media as in the columns including pesticides, DOC and electron acceptor (Fe(III) or sulfate) following protocols from Luo et al. 2019. Fe(III) oxides were produced by neutralizing a 0.4 M FeCl_3_ solution with NaOH until reaching pH 7. In order to remove the dissolved chloride, solution was centrifuged, and solids were further re-suspended in demi-water before use. The headspace of the enrichment microcosms was filled with N_2_ gas. The mixed enrichments were kept on a shaker operated at 120 rpm and 30 °C. After several transfers of the original microcosms to fresh media, the mixed enrichments with the ability to degrade at least one of the pesticides (i.e., 2,4-D) were selected (Figure S1). The final two inocula (one per redox conditions) were created by combining the triplicate microcosms from the same redox condition in an anoxic bottle before inoculation.

##### Bioaugmentation inoculation

The columns were bioaugmented on two occasions. At days 219 (column1) and 220 (column2) the first bioaugmentation (BA1) took place. At days 583 (column1) and 600 (column2) the second bioaugmentation (BA2) was conducted. The columns were inoculated with a mixed enrichment representing 10% of the total column fluid volume, namely 132 mL for column1 and 148 mL for column2. The differences in column liquid volume correspond to differences in the sediment height per column. Together with the enrichment, a fluorescein solution (10 mg/L) was used as an inert tracer of the inoculated liquid in both columns during BA1. A sterile syringe with a volume of 150 mL and a syringe pump (Kd Scientific, USA) were utilized to bioaugment on two occasions each column at SP_2_ using an injection rate of 0.14 mL/min, similar to the medium flow injection rate (8 mL/h). The injection rate resulted in an inoculation time of almost 16 h for column1 and almost 18 h for column2.

##### Sampling

During the column operation (~ 800 days), media and SP_out_ liquid samples (2 mL) were collected and centrifuged at 15.000 rpm for 10 min and stored at − 20 °C before pesticide quantification (Sect. “Pesticide quantification”). After BA1, fluorescein concentration was measured in SP_1_, SP_2_, SP_3_ and SP_out_ until the concentration in the outlet was below the detection limit (Sect. “Fluorescein measurements”). Additionally, liquid (~ 4 mL) and sediment (~ 2.5 g) samples were collected from SP_2_ and SP_out_ at every time point for microbiological analyses as well as samples from the inocula added in BA1 and BA2 (Sect. “Microbiological analysis”) as presented in Table 1.

**Table 1:** Sampling days for microbiological analyses according to the experimental phase

	Column1	Column2
Inocula1	218	219
Before BA	219	220
After BA1	269	277
	311	322
	583	592
Inocula2	582	591
After BA2	599	615
	775	795

### Sample analysis

#### DOC concentration

DOC concentration of the stock solution was determined by measuring NPOC (non-purgeable organic carbon) with a TOC-L_CPH_ analyser with an ASI-L autosampler (Shimadzu, Japan). The final DOC concentration per medium bottle was adjusted to 10 mgC/L.

#### Pesticide quantification

BAM, MCPP and 2,4-D were quantified with a UPLC-DAD (Ultra high performance liquid chromatography with diode array detection) system. The eluents used for this method were ultra-pure water with 0.1% formic acid (FA) and acetonitrile with 0.1% FA. Details of the method can be found in Luo et al. (2019). The removal efficiency of each pesticide was calculated using Eq. 1, where *C*_*media*_ is an average from the first and last day (around 14 days) of each media bottle to adjust for changes in pesticide concentration in the medium bottles.

1 $$  Removal{\text{ }}efficiency{\text{ }}\left( \%  \right) = \frac{{C_{{media}}  - C_{{out}} }}{{C_{{media}} }} \times 100$$

  

#### Fluorescein measurements

The absorbances from the samples collected were measured at 486 nm using a Xion 500 spectrophotometer (Dr. Bruno Lange GmbH & Co., Berlin, Germany). The fluorescein concentration were calculated based on calibration curves that were prepared separately for each experimental day.

#### Microbiological analysis

##### DNA extraction, quantification and sequencing

Microbial DNA was extracted from liquid and sediment samples using the DNeasy PowerSoil Kit (Qiagen, Hilden, Germany) according to the manufacturer’s instructions. DNA was quantified using the dsDNA HS Assay kit for Qubit fluorometer (Invitrogen, Grand Island, NY). Preparation of the sequencing library was conducted following a previous study (Ramiro-Garcia et al. 2018). The targeting region was V4, and primers 515F (GTGYCAGCMGCCGCGGTAA) and 806R (GGACTACNVGGGTWTCTAAT) were used (Walters et al. 2015). In short, the PCR mix was prepared as described in Table S1, and the PCR program as detailed in Table S2. The PCR products were cleaned with the MagBio Beads Cleanup Kit (MagBio) according to the manufacturer’s instructions. Quality and concentration of the PCR products was checked with 1% (w/v) agarose gels stained with 1× SYBR® Safe (Invitrogen) and quantified using Qubit as described above. Samples were barcoded and pooled in equimolar concentrations (4 × 10^6^ copies/µL) and sent for sequencing on an Illumina System, Paired-end 150 bp (Novogene Europe). To ensure high quality sequencing data, each sequence library contained two synthetic Mock communities of known composition that were used as positive controls (Ramiro-Garcia et al. 2018), and nuclease free water as negative controls. The sequenced Mock communities resulted in a Pearson correlation range with the theoretical Mock composition of 0.86–0.96. The negative controls contained 3 and 82 reads, while the sample datasets contained at least 10577 reads. This indicated high quality of the sequence data. Sequence data was submitted to the European Bioinformatics Institute under study accession No. PRJEB42809 (ERP126720). The list of samples and barcodes is provided in Table S3.

## Sequencing data processing and analysis

The 16S rRNA gene sequence data was analysed using NG-tax 2.0 (Poncheewin et al. 2020). Briefly, to generate amplicon sequence variants (ASVs), NG-Tax 2.0 employs a de novo ASV-picking algorithm. To assign taxonomy the SILVA 132 16 S rRNA gene reference database was used (Quast et al. 2013). The phylogenetic tree was created with Clustal Omega (Sievers et al. 2011). The ASVs associated with an unknown domain, the kingdom Archaea, the family Mitochondria or the order Chloroplast were removed from all sequenced samples. All statistical analyses were performed in R version 4.0.2 using the packages: Phyloseq, Microbiome and Vegan (Lahti and Shetty 2017; Mcmurdie and Holmes 2013; Oksanen et al. 2016). Beta diversity (divergence in microbiota composition between samples) was determined using unweighted UniFrac (UF) distance metrics (Lozupone and Knight 2008). The variation between samples was visualized using principal coordinates analysis (PCoA). All R-scripts, data files and pdf files with extensive information on the performed analyses can be accessed through the Github page: https://github.com/mibwurrepo/Aldas-Vargas_etal_Bioaugmentation.

### qPCR analysis

qPCR (quantitative polymerase chain reaction) analyses were used to quantify the 2,4-D aerobic degradation (*tfdA*) (Bælum et al. 2008). Analyses were performed on an iQ SYBR Green using Bio-Rad super mix in combination with a CFX384 Touch™ Real-Time PCR Detection System. All qPCR assays were performed in triplicate with a reaction volume of 10 µL. Gene copy numbers were calculated per mL (liquid) or per g (sediment) depending on the sample. Detailed information of the qPCR primers and amplification protocols can be found in Table S4.

## Results and discussion

### Biodegradation activity prior to BA

**Fig. 2:**
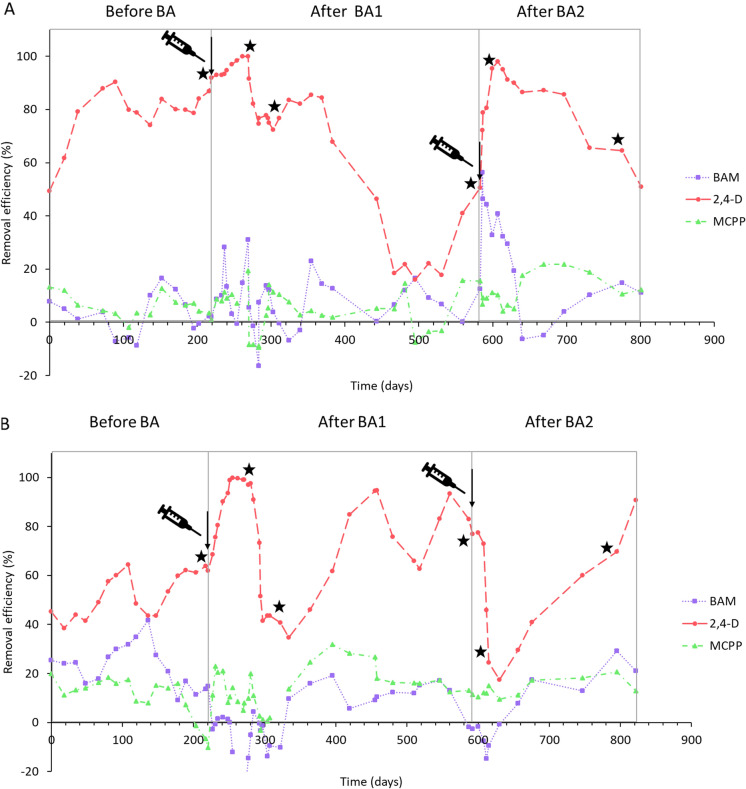
Removal efficiencies of BAM, 2,4-D and MCPP for column1 (**A**) and for column2 (**B**) before and after the BA inoculation. The stars represent sampling for microbiological analysis according to Table 1. The syringes and vertical arrows indicate BA inoculation events

The removal efficiencies of BAM, 2,4-D and MCPP were monitored for around 800 days, before, during and after two rounds of BA (Fig. 2). Before BA, pesticide removal was monitored to determine biodegradation activity of the simulated groundwater systems. In the case of BAM and MCPP there was no significant degradation observed. Due to the chemical-structure similarities between 2,4-D and MCPP, it has been reported that microorganisms can degrade both pesticides (Toräng et al. 2003). In that study, the main pre-requisite was that samples had been pre-exposed to both compounds. The importance of exposure for the development of MCPP biodegradation has been highlighted for phenoxy-acid pre-exposed soils (Frková et al. 2016). In our study, we did not observe MCPP degradation in either of the two columns, even though we observed 2,4-D removal and even though microorganisms in the columns had been exposed for a long period of time (~ 800 days).

Prior to BA, 2,4-D removal was observed in both column reactors. The 2,4-D removal efficiencies in percentage fluctuated from 75 ± 14 to 82 ± 5% in column1, and from 50 ± 9 to 55 ± 8% in column2 (Table 2). In a previous study where different amendments were tested in groundwater samples for the biodegradation of BAM, 2,4-D and MCPP, it was observed that 2,4-D was the more easily degradable compound (Aldas-Vargas et al. 2021). The three compounds were tested together to simulate groundwater conditions, where multiple micropollutants are present simultaneously (Loos et al. 2010; Sjerps et al. 2019). Yet, it is possible that 2,4-D is preferentially degraded and its presence thus limits the biodegradation of the other two pesticides.

Under iron-reducing conditions (column1), the pre-BA 2,4-D removal efficiencies were higher than under sulfate-reducing conditions (column2) (Fig. 2). Literature studies showed that there are some factors that can result in a better 2,4-D degradation under iron-reducing conditions, for instance that: (a) bacteria can obtain more energy from iron than from sulphate reduction (Chapelle and Lovley 1992), and that (b) there are enzymes involved in 2,4-D degradation that are iron dependent (Jia et al. 2017). Although there are a couple of proposed anaerobic 2,4-D biodegradation pathways (Brucha et al. 2021; Ha 2018), genes associated with anaerobic biodegradation have not yet been identified and therefore not feasible to monitor in our experiment. However, we did assay the *tfdA* gene, identified in the aerobic biodegradation of 2,4-D. In the column samples, *tfdA* was present in SP_2_ sediment in higher abundance for column1 compared to column2 (Figure S2), which is in line with our observation of a higher 2,4-D removal efficiency for column1 (Fig. 2). However, it is questionable whether this can be directly used as an indication of 2,4-D biodegradation capacity. This, because the *tfdA* gene is an indicator of aerobic 2,4-D biodegradation pathways, which are not expected under the iron-reducing conditions from column1 or sulfate-reducing conditions from column2.

**Table 2:** Summary of removal efficiencies around every 100 days for the phases: Before BA, After BA1 and After BA2 for columns 1 and 2

Removal efficiencies (%)								
Phase	Days	Column1			Days	Column2		
		BAM	2,4-D	MCPP		BAM	2,4-D	MCPP
Before BA	0 to 118	− 0.5 ± 6.1	75.4 ± 13.6	5.8 ± 4.9	0 to 108	24.5 ± 5.1	50.0 ± 8.9	15.8 ± 2.7
	136 to 219	5.9 ± 6.2	82.4 ± 5.1	5.7 ± 3.4	119 to 220	21.2 ± 10.0	55.4 ± 7.8	5.6 ± 9.5
After BA1	226 to 311	8.6 ± 10.8	86.7 ± 10.0	5.2 ± 8.8	227 to 322	− 5.7 ± 8.6	78.2 ± 22.4	9.7 ± 7.5
	324 to 443	6.8 ± 10.6	75.0 ± 14.1	4.1 ± 2.0	334 to 458	11.7 ± 4.5	69.5 ± 23.5	23.8 ± 6.2
	467 to 583	9.1 ± 4.8	26.8 ± 12.4	5.3 ± 9.3	480 to 592	9.3 ± 7.5	77.3 ± 9.8	14.7 ± 2.1
After BA2	586 to 669	29.1 ± 19.8	87.5 ± 7.8	10.2 ± 5.3	600 to 676	− 1.3 ± 10.1	44.1 ± 21.6	12.5 ± 2.5
	697 to 801	10.1 ± 3.9	66.7 ± 12.4	15.9 ± 4.7	747 to 822	21.0 ± 6.6	73.5 ± 12.8	17.2 ± 3.2

Columns were continuously operated and data is based on sampling dates. Removal efficiencies are the averages of the data points around every 100 days and standard deviations represent the fluctuations among the different data points

### Biodegradation activity after BA1

BA with a 2,4-D degrading mixed enrichment culture resulted in improved 2,4-D removal efficiencies in both columns, however the impact was temporary (Fig. 2). There was no notable influence on the removal efficiencies of BAM and MCPP in the columns after BA1, which was expected since the batch enrichments did not show the capability to biodegrade the aforementioned pesticides (Figure S1). It should be noted that BA did not result in measurable pesticide dilution, which is confirmed by the lack of changes in the removal efficiency of BAM and MCPP (Fig. 2). For column1, the 2,4-D removal efficiencies were already above 75% before BA. However, removal to below the detection limit was achieved at day 261 after the BA inoculation (Fig. 2). The positive effect of BA1 in column1 lasted around 100 days (from day 226 to 311), with removal efficiency increasing to 87 ± 10% (Table 2). After some time, a significant drop in 2,4-D removal efficiency was observed from days 467 to 583 to 27 ± 12% (Table 2). In column2, BA resulted in an increase in 2,4-D removal to below detection limit on day 255 (Fig. 2). The 2,4-D removal efficiency increased to 78 ± 22% from days 277 to 322. On day 334 the lowest 2,4-D removal efficiency was observed with around 34%. Later on, the 2,4-D removal efficiency increased and was maintained in the period 480 to 592 days (77 ± 10%) (Table 2). The positive effect of BA1 lasted around 16 days longer in column2 than in column1. The major reasons for this observation may be that before BA column1 had already 2,4-D removal efficiencies above 80%, while column2 had mainly removal efficiencies of 50%. This means that in column1 there were already microbial communities with high 2,4-D removal capabilities compared to column2. Thus, when adding the BA inocula, also with 2,4-D degradation activity, there was probably more microbial competition in column1 compared to column2.

To follow inocula distribution, inocula were mixed with a fluorescein tracer prior to injection. Fluorescein concentration was evaluated at different sampling points. Fluorescein was detected in SP_1_ and SP_3_ almost immediately after injection, showing that the inocula spread well along the column. Later, fluorescein was detected in both columns after 21 days, much longer than the residence time of 6.8 days for column1 and 7.70 days for column2 (Figure S3), indicating that there is liquid retention in both columns. If liquid was retained longer in the columns, it can also be assumed that the inoculated microbial communities stayed in the column for a longer time than the residence time.

**Fig. 3:**
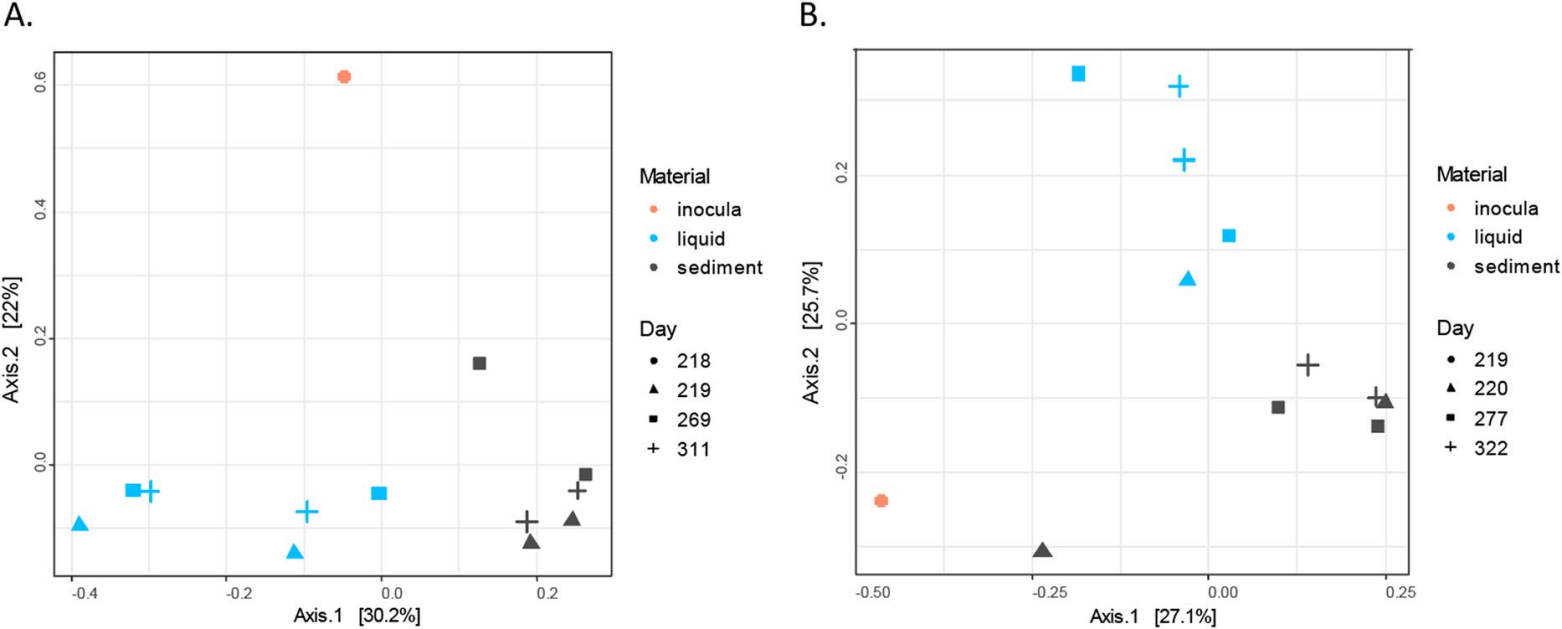
Principle coordinate analysis (PCoA) of microbial communities based on pairwise unweighted Unifrac distances before and after BA1, for **A** column1 and **B** column2. Different colors indicate different sampling materials, with circles for inocula, triangles are sampling time point 2, squares are sampling time point 3, and cross are sampling time point 4

The influence of BA was also observed in microbial community composition. There was a clear distinction between liquid and sediment samples (Fig. 3), which indicates that both planktonic and biofilm forming microorganisms were present in the columns. Furthermore, when looking at the differences in microbial composition from the column samples before and after BA1, we observed that at days 269 (column1) and 277 (column2), the microbial composition shifted in the sediment samples from SP_2_, compared to days 219 (column1) and 220 (column2) before BA1 (arrow, Fig. 3). This shift suggests an influence of the inocula on microbial community composition. However, this shift was temporary. At days 311 (column1) and 322 (column2), the microbial composition of the column samples had returned back to their initial composition prior to BA. This means that any subsequent degradation after days 311 and 322, would be potentially conducted by the column autochthonous community. The observed changes in microbial composition coincided with 2,4-D removal efficiencies. For instance, the highest peak in 2,4-D removal efficiency for column1 (Fig. 2) occurred the same time (day 269), as when the SP_2_ sediment samples are more similar to the inocula (Fig. 3A). In column2, the initial microbial composition of the column was already similar to the inoculum, but at the highest 2,4-D degradation rates (day 277), the microbial composition is also more similar to the inoculum compared to day 322, where removal efficiencies decreased. These results suggest that the inocula addition had a positive effect in the 2,4-D removal efficiency for both columns.

### Biodegradation activity after BA2

**Fig. 4:**
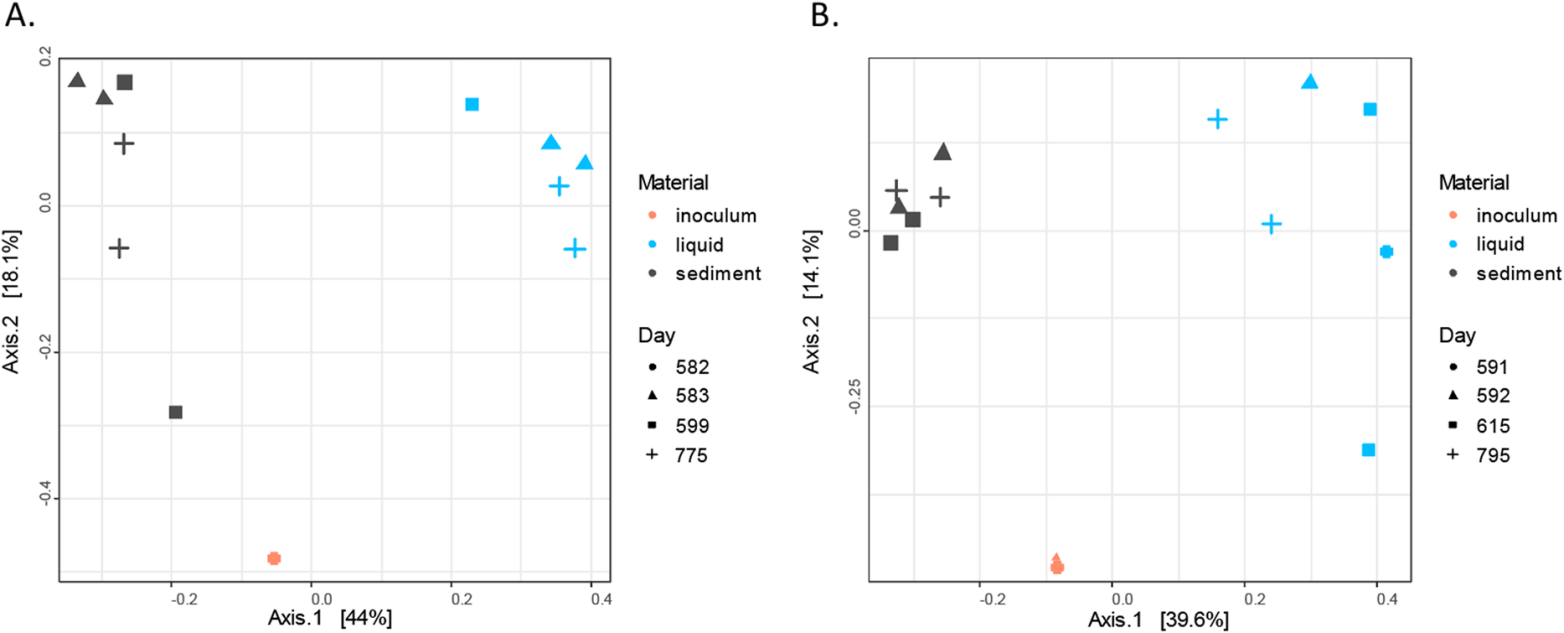
Principle coordinate analysis of microbial communities based on unweighted Unifrac before and after BA1, for **A** column1 and **B** column2. Different colors indicate different sampling materials, circles inocula samples, triangles are sampling time point 2, squares are sampling time point 3, plus are sampling time point 4

BA2 resulted in an increase in 2,4-D removal in column1, and a decrease in 2,4-D removal in column2 (Fig. 2). In column1, the average 2,4-D removal efficiency after 30 days increased up to 95% after BA2 (day 614, Fig. 2). Afterwards the 2,4-D removal efficiency slowly decreased to 50% by the end of the experiment (day 801, Fig. 2). In column2, the 2,4-D removal decreased to 17% after 30 days of BA2 (day 630, Fig. 2). Afterwards, the 2,4-D removal efficiency slowly increased again up to 90% by the end of the experiment (day 822, Fig. 2). In both cases, the effect of BA2 was temporary, similar to what was observed for BA1. A clear distinction between the liquid and the sediment samples was once again observed as in BA1 (Fig. 4). At day 599, after BA2, we observed in column1 that the microbial community in the SP_2_ sediment sample was more similar to the inoculum than prior to inoculation (Fig. 4A). This shift in microbial communities coincided with an increase in 2,4-D removal efficiency, suggesting that increased 2,4-D removal is related to BA2. For column2, microbial composition remained stable and did not reflect any influence from the BA2 inoculum (Fig. 4B). However, a decrease in removal efficiency of 2,4-D from 77 ± 10, to 44 ± 22% after BA2 was observed (Table 2). In that sense, even if the inoculum did not clearly change the microbial composition, there was a negative effect in terms of 2,4-D removal efficiency.

**Fig. 5:**
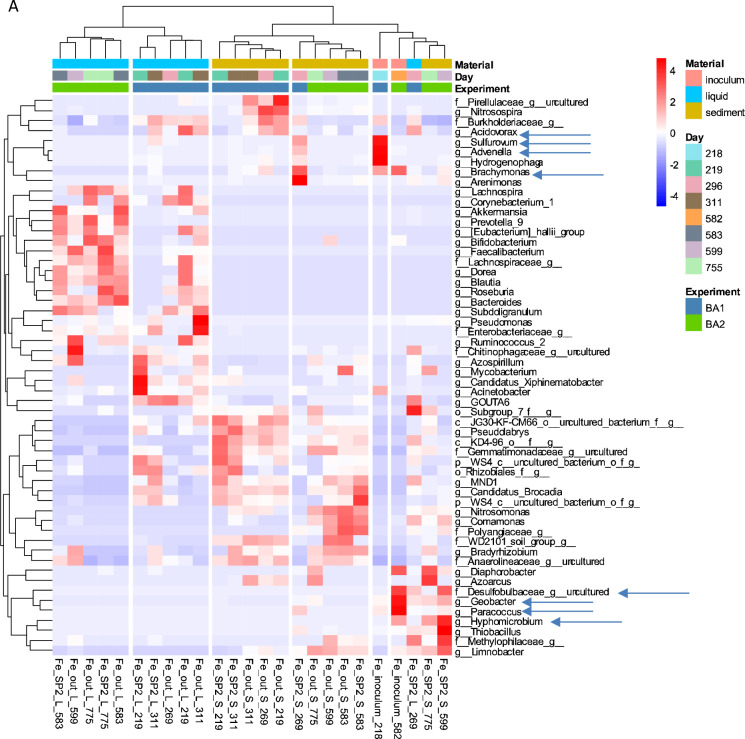

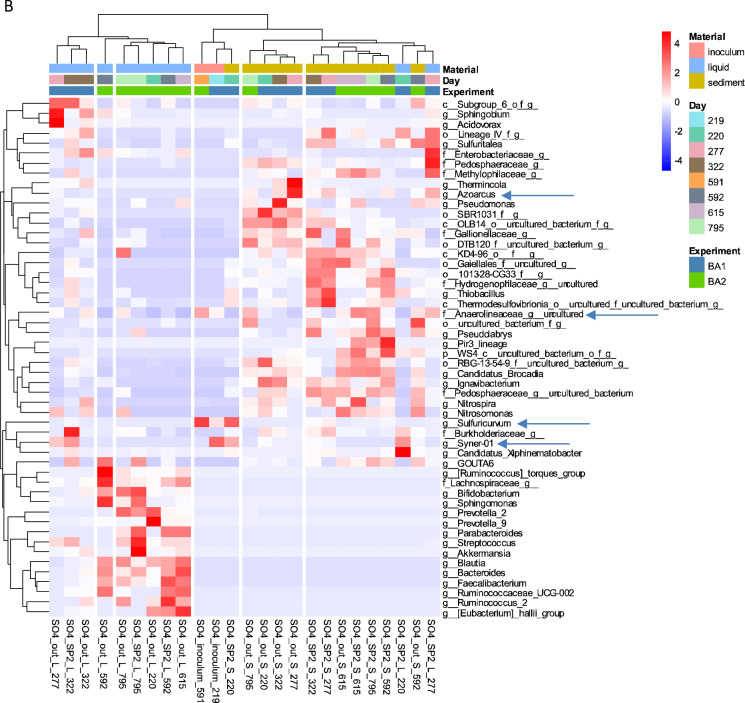
Heatmap of main genera with relative abundance ≥ 0.01% and prevalence ≥ 10% in column1 (**A**) and column2 (**B**). Clustering of samples is based on Ward’s minimum variance method and based on unweighted UniFrac distances matrix. The six clusters are presented in the figure (n = 25 per column)

We observed that the effect of BA1 and BA2 in terms of removal efficiency and microbial community composition was temporary (Figs. 4 and 5), which is something previously reported in literature (Bouchez et al. 2000; Dai et al. 2015; Ellegaard-Jensen et al. 2020; Wenderoth et al. 2003). There are different possibilities for the temporary effect of the BA, such as: (a) survival of the inocula, (b) wash out of the inocula and (c) loss of degradation activity. In our experiment, BA took place in a closed system in which the autochthonous community is fully adapted to the environment and may outcompete the added inocula, as observed in a similar study (Bouchez et al. 2000). However, the inocula initially survived, resulting in a temporarily and reversible shift in the microbial composition, as reflected in the Unifrac results (Figs. 4 and 5). As to the system wash out (b), we intended to prevent this from happening by having a rather low injection flow rate in both inoculations, which was the same as the media injection flow (0.14 mL/min). A lower injection flow guaranteed that the inocula could distribute well in the column (Figure S3) during the 16–18 h that inoculation took. We observed that microbial communities from sediment and liquid samples differ. However, both planktonic and biofilm forming bacteria may have stayed in the columns due to the dead space demonstrated by fluorescein data. With respect to the last possible explanation (c), microorganisms retained in the system can lose their biodegradation activity because of nutrient limitations (Albers et al. 2014). It has been previously reported that even if the added inoculum is retained in the system, pesticide biodegradation activity can be lost in time (Ellegaard-Jensen et al. 2020). In our system, we have evidence that the effect of BA affected temporarily the microbial composition. Thus, a loss of activity is less likely since the added inocula was not retained for a long time as shown in Figs. 3 and 4. Besides, nutrient limitation in the columns was also discarded because they were continuously fed with fresh medium.

### Microbial communities dynamics

The temporary effect of BA in 2,4-D removal coincides with the temporary change of microbial composition in SP_2_ sediment samples after BA1 and BA2 for column1 and after BA1 for column 2 (Figs. 3 and 4). The microbial composition of the columns was further explored looking at changes in the relative abundance of particular microbial groups at the genus level after BA (Fig. 5). In column1, after BA1, the genera that were found in the SP_2_ sediment samples at day 269 that coincided with those observed in the inocula were *Sulfurovum, Advenella*, and *Brachymonas* (Fig. 5A). Similarly, after BA2, *Geobacter, Paracoccus* and *Hyphomicrobium* were found in both the inoculum and the SP_2_ sediment sample at day 599 (Fig. 5A). In column2, after BA1 there was an increase in the relative abundance of genus *Syner-01* from the *Synergistaceae* family in the SP_2_ sediment samples at day 277 (Fig. 5B). In that sense, those changes in relative abundance of the above mentioned microorganisms, indicate that they might have entered the column via the inocula, and that they can include some of the responsible groups for the similarities between inocula and SP_2_ sediment samples displayed before (Figs. 4 and 5).

The dichotomy between liquid and sediment samples was previously observed for both columns (Figs. 3 and 4). There was also a clear distinction in the relative abundance of microbial groups in the sediment and liquid samples (Fig. 5). It is for instance obvious that the predominant planktonic taxa were less present in sediments and the other way around (Fig. 5). In terms of BA, microorganisms that could attach to sediments can have lower risk of wash out. The attachment or not of bacteria to the sediments, was reported to depend on microbial as well as on sediment characteristics (Cápiro et al. 2014). Some of the microbial communities present for instance in the SP_2_ sediment samples at day 269 were not present anymore at day 311 (Fig. 5A). In contrast, there were more microbial groups shared in the SP_2_ sediment samples at days 277 and 322 (Fig. 5B). Examples of these microbial groups are: *Gaiellales, Thiobacillus, Thermodesulfovibriona*, among others. Although these microbial groups may have stayed longer in the system, there is currently no evidence for their contribution for the 2,4-D removal efficiency. This, especially considering that the 2,4-D removal efficiencies for column2 after BA1 showed a decrease from around 100% at day 277 compared to around 40% at day 322 (Table 2).

There were some known contaminant degraders present in the columns (Figure. 5A, B). For instance, *Acidovorax*, which was present in column1 in the BA1 inoculum as well as in the SP_2_ sediment samples at day 269 has been recognized as a degrader of halogenated compounds including herbicides (Pileggi et al. 2020; Singleton et al. 2009, 2018). Furthermore, *Azoarcus*, which was present in column2 in the sediment samples SP_2_ and out at day 277 (Fig. 5B), was previously found in groundwater systems and associated with contaminant degradation (Griebler and Lueders 2009; Héry et al. 2014). More interestingly, *Azoarcus* showed to be able to degrade anaerobically a significant number of aromatic compounds, including benzoate which contains a benzene ring similar to 2,4-D (Fernández et al. 2014; López Barragán et al. 2004). Furthermore, the species *Azoarcus sp*. KH32C was reported to contain a 2-oxoglutarate-dependent taurine catabolism dioxygenase (TauD), which belongs to the TauD/TfdA dioxygenase family (Uniprot 2021), and which was also used in this study for *tfdA*-targeted monitoring 2,4-D degradation activity. In our study, *Azoarcus* relative abundance (Fig. 5B) coincided with an increase in the 2,4-D degradation (Fig. 2). However, further research is necessary to be able to confirm the link between the aforementioned microbial groups and changes in pesticide biodegradation.

### Recommendations and implications for field application of bioaugmentation

BA showed to have a temporary effect in the removal efficiency of pesticides. We discussed before some possibilities for having a temporal degradation effect, namely: (a) survival of the inocula, (b) wash out of the inocula and (c) loss of degradation activity. In the field, it is less likely that there is a system washout occurring, but there is the possibility that the inocula does not meet the contaminant due to field heterogeneity. The survival of the inocula is related with the selection of suitable microbial communities, which have been pre-exposed to contaminants, and adapted to environmental field conditions. The loss of degradation activity has been related with the lack of nutrients, which in a oligotrophic environment like groundwater may occur. Furthermore, field monitoring of pesticide degradation is in general complex, due to heterogeneously distributed pesticides, changing geochemical conditions and also lack of effective monitoring tools for field microbial communities (Aldas-Vargas et al. 2022b). Therefore, tracking inocula survival or activity might not be feasible. Despite that, for real field application, the periodic addition of a degrading inoculum would be necessary to overcome the BA temporary effect observed in this experiment and to achieve the desired pesticide removal.

BA as remediation technology faces several challenges that were encountered in the present study. On one hand, the application of a mixed inoculum instead of a single strain can support microbial dynamics that enhance pesticide biodegradation. On the other hand, the addition of a mixed inoculum impedes straight forward monitoring of the resilience of the BA community and may mask certain microbial processes. Furthermore, the demonstration that iron-reducing conditions resulted in overall a higher 2,4-D degradation activity compared to sulfate reducing conditions shows that BA may be more successful in certain depths of the aquifer depending on its redox condition. Thus, the question of BA suitability for all the aquifer depths requires further research. Additionally, due to the temporary impact of BA, more than one intervention may be necessary, which could require constant need for addition of an active inoculum or other amendments. Finally, it is difficult to predict what the long term effect of BA is on biomass presence in aquifers, and thus the impact of BA on current drinking water production processes. Overall, it is clear that further research is required to improve BA for the bioremediation of pesticides in drinking water aquifers.

Our results showed that the outcome of BA is sometimes unpredictable (column2, BA2). This happens because there are many microbial ecology dynamics happening that unfortunately we cannot fully track. Although we monitored the different microbial groups present in the column, it still unknown which microbial interaction resulted in the increase or decrease in relative abundance of certain groups. For instance, it is not clear if there is cooperation, predation or competition between the added inocula and the column microorganisms. Furthermore, additional research is necessary to better understand if the added inocula catabolizes the pesticides present in the system. For this, next to changes in pesticides concentration, also the ^12^C/^13^C ratios can be utilized to compare pesticide’s microbial consumption before and after BA (Elsner and Imfeld 2016). In this study, two redox conditions were tested, but in groundwater systems there are more redox that also need to be investigated. Likewise, the BA inoculum can be characterized not only in terms of microbial composition but also in terms of metabolic capacity by the use of metagenomics. Metagenomics can not only contribute to inoculum characterization but also to the development of target genes from anaerobic degradation pathways that facilitate pesticides’s biodegradation future monitoring.

## Conclusion

We studied the effects of BA with mixed inocula on anaerobic pesticide biodegradation in column bioreactors that simulated groundwater systems at different redox conditions. The effects of BA were evaluated in terms of removal efficiency of BAM, MCPP and 2,4-D and the microbial community dynamics were studied before and after inoculation. We observed that 2,4-D removal efficiency had a temporary change after the addition of a 2,4-D degrading mixed inocula. Moreover, BA had a temporary effect on the microbial composition of the simulated groundwater systems. Our results suggest that pesticide removal efficiency can increase by the addition of a degrading mixed inocula, and that iron-reducing conditions are more favourable for 2,4-D degradation than sulfate-reducing conditions. The present study demonstrates that BA is a valuable technique for improving 2,4-D biodegradation in stable systems. For future studies, enrichments from different sources may be tested to determine if different microbial community sources and compositions result in improved BA. Also, a combination of biostimulation and bioaugmentation can be studied in a continuous system, as results indicate that this will be necessary under field conditions. Finally, BA potential for the removal of other persistent pesticides should be tested as well as different strategies for scaling-up this technology towards in-situ field application.

### Supplementary Information

Below is the link to the electronic supplementary material.

Supplementary material 1 (DOCX 123.1 kb)
