# Supplementary material for: Bioaugmentation has temporary effect on anaerobic pesticide biodegradation in simulated groundwater systems
Source: Biodegradation. 2023 Jul 13;35(3):281–97. doi: 10.1007/s10532-023-10039-0 (PMC10951022; doi:10.1007/s10532-023-10039-0)
Supplement: Supplementary file 1 — Supplementary material 1 (DOCX 123.1 kb) [file 10532_2023_10039_MOESM1_ESM.docx]

Table S1. Preparation of PCR mastermix for samples

| Component | Volume (µl) |
| --- | --- |
| 5x HF buffer (green) | 7 |
| Primer 515F - 1, 806R – 1 (10 µM)  515F – 1, 806R – 1  Etc, until barcode 70 | 0.7 |
| dNTPs | 0.7 |
| Phusion Hot start II DNA polymerase (2U/µl) | 0.35 |
| Nuclease free water | 25.5 |
| Total | 34.3ul |
| DNA template (20ng/ul) | 0.7 |

* Primers were prepared by mixing 10 µl of primer 515F (100 µM) and 10 µl of 806R (100 µM) with 80 µl nuclease free water.

Table S2. Amplification program for the PCR used for samples

|  | Temperature (°C) | Time |
| --- | --- | --- |
|  | 98 | 30 seconds |
| 25 cycles | 98 | 10 seconds |
|  | 50 | 10 seconds |
|  | 72 | 10 seconds |
|  | 72 | 7 minutes |
|  | 12 | ∞ |

Table S3. Barcode sequences for each sample used in MiSeq sequencing

| sample_alias | sample_name | forwardBarcode | reverseBarcode | Material | Day | Redox |
| --- | --- | --- | --- | --- | --- | --- |
| 2020_05599 | Fe_ino_218 | CTTGCGAG | CTTGCGAG | inoculum | 218 | Fe |
| 2020_05601 | Fe_SP2_L_219 | GAACCGTT | GAACCGTT | liquid | 219 | Fe |
| 2020_05605 | Fe_SP2_S_219 | GAGTTATA | GAGTTATA | sediment | 219 | Fe |
| 2020_05613 | Fe_out_L_219 | GGAGTATG | GGAGTATG | liquid | 219 | Fe |
| 2020_05623 | Fe_out_S_219 | TCCTCCGC | TCCTCCGC | sediment | 219 | Fe |
| 2020_05615 | Fe_SP2_L_269 | GGTAGAAT | GGTAGAAT | liquid | 269 | Fe |
| 2020_05621 | Fe_SP2_S_269 | TCATTCCG | TCATTCCG | sediment | 269 | Fe |
| 2020_05627 | Fe_out_L_269 | TGACTCAA | TGACTCAA | liquid | 269 | Fe |
| 2020_05617 | Fe_out_S_269 | GTTAAGTT | GTTAAGTT | sediment | 269 | Fe |
| 2020_05631 | Fe_SP2_L_311 | TGGTATGA | TGGTATGA | liquid | 311 | Fe |
| 2020_05597 | Fe_SP2_S_311 | AACCATGC | AACCATGC | sediment | 311 | Fe |
| 2020_05750 | Fe_out_L_311 | GGTAGAAT | GGTAGAAT | liquid | 311 | Fe |
| 2020_05607 | Fe_out_S_311 | GATGATAA | GATGATAA | sediment | 311 | Fe |
| 2020_05611 | Fe_ino_582 | GCTAATCT | GCTAATCT | inoculum | 582 | Fe |
| 2020_05603 | Fe_SP2_L_583 | GAACTAAG | GAACTAAG | liquid | 583 | Fe |
| 2020_05754 | Fe_SP2_S_583 | TATTGCGC | TATTGCGC | sediment | 583 | Fe |
| 2020_05758 | Fe_out_L_583 | TCCTCCGC | TCCTCCGC | liquid | 583 | Fe |
| 2020_05609 | Fe_out_S_583 | GCCAGGTT | GCCAGGTT | sediment | 583 | Fe |
| 2020_05790 | Fe_SP2_L_599 | CATAAGCG | CATAAGCG | liquid | 599 | Fe |
| 2020_05788 | Fe_SP2_S_599 | CAACCTCT | CAACCTCT | sediment | 599 | Fe |
| 2020_05629 | Fe_out_L_599 | TGCCTGCG | TGCCTGCG | liquid | 599 | Fe |
| 2020_05619 | Fe_out_S_599 | TATTGCGC | TATTGCGC | sediment | 599 | Fe |
| 2020_05756 | Fe_SP2_L_775 | TCATTCCG | TCATTCCG | liquid | 775 | Fe |
| 2020_05752 | Fe_SP2_S_775 | GTTAAGTT | GTTAAGTT | sediment | 775 | Fe |
| 2020_05633 | Fe_out_L_775 | TTGACTAG | TTGACTAG | liquid | 775 | Fe |
| 2020_05625 | Fe_out_S_775 | TCGATATT | TCGATATT | sediment | 775 | Fe |
| 2020_05600 | SO4_ino_219 | CTTGGCCT | CTTGGCCT | inoculum | 219 | SO4 |
| 2020_05602 | SO4_SP2_L_220 | GAACGTAT | GAACGTAT | liquid | 220 | SO4 |
| 2020_05606 | SO4_SP2_S_220 | GATGAATG | GATGAATG | sediment | 220 | SO4 |
| 2020_05614 | SO4_out_L_220 | GGTACCAA | GGTACCAA | liquid | 220 | SO4 |
| 2020_05624 | SO4_out_S_220 | TCGAATAA | TCGAATAA | sediment | 220 | SO4 |
| 2020_05616 | SO4_SP2_L_277 | GTCCGCAA | GTCCGCAA | liquid | 277 | SO4 |
| 2020_05622 | SO4_SP2_S_277 | TCCGTATA | TCCGTATA | sediment | 277 | SO4 |
| 2020_05628 | SO4_out_L_277 | TGATCTCA | TGATCTCA | liquid | 277 | SO4 |
| 2020_05618 | SO4_out_S_277 | GTTCTACG | GTTCTACG | sediment | 277 | SO4 |
| 2020_05632 | SO4_SP2_L_322 | TTAGGATG | TTAGGATG | liquid | 322 | SO4 |
| 2020_05598 | SO4_SP2_S_322 | ACCGGAAT | ACCGGAAT | sediment | 322 | SO4 |
| 2020_05751 | SO4_out_L_322 | GTCCGCAA | GTCCGCAA | liquid | 322 | SO4 |
| 2020_05608 | SO4_out_S_322 | GATGCGCT | GATGCGCT | sediment | 322 | SO4 |
| 2020_05612 | SO4_ino_591 | GGAGCGCA | GGAGCGCA | inoculum | 591 | SO4 |
| 2020_05604 | SO4_SP2_L_592 | GAAGCTCG | GAAGCTCG | liquid | 592 | SO4 |
| 2020_05755 | SO4_SP2_S_592 | TCAGCGAG | TCAGCGAG | sediment | 592 | SO4 |
| 2020_05759 | SO4_out_L_592 | TCGAATAA | TCGAATAA | liquid | 592 | SO4 |
| 2020_05610 | SO4_out_S_592 | GCCTTAAG | GCCTTAAG | sediment | 592 | SO4 |
| 2020_05791 | SO4_SP2_L_615 | CATGATGC | CATGATGC | liquid | 615 | SO4 |
| 2020_05789 | SO4_SP2_S_615 | CAACGAGG | CAACGAGG | sediment | 615 | SO4 |
| 2020_05630 | SO4_out_L_615 | TGGTAATT | TGGTAATT | liquid | 615 | SO4 |
| 2020_05620 | SO4_out_S_615 | TCAGCGAG | TCAGCGAG | sediment | 615 | SO4 |
| 2020_05757 | SO4_SP2_L_795 | TCCGTATA | TCCGTATA | liquid | 795 | SO4 |
| 2020_05753 | SO4_SP2_S_795 | GTTCTACG | GTTCTACG | sediment | 795 | SO4 |
| 2020_05634 | SO4_out_L_795 | TTGATCCA | TTGATCCA | liquid | 795 | SO4 |
| 2020_05626 | SO4_out_S_795 | TCTATTCG | TCTATTCG | sediment | 795 | SO4 |

Table S4. Target genes, primer sequence and amplification program used for quantification of the functional genes

| Target gene | Primer names (if applicable) and oligonucleotide sequence | Thermal profile | Cycles | Ref. |
| --- | --- | --- | --- | --- |
| *tfdA* | F: (5’-GAGCACTACGC(AG)CTG AA(CT)TCCCG-3’ )  R: (5’-GTCGCGTGCAGAAG-3’) | 95°C 10 min  95°C 30 s, 60°C 30 s, 72°C 30 s | 1  46 | (Bælum et al., 2008) |

| **A** | **B** |
| --- | --- |
| **C** | **D** |
| **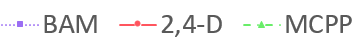** | |

**Figure S1. Averages of residual pesticide concentration in enrichment triplicate batch bottles used for different bioaugmentation experiments. A) For column 1 BA1; B) For column 2 BA1; C) For column 1 BA2; D) For column 2 BA2**


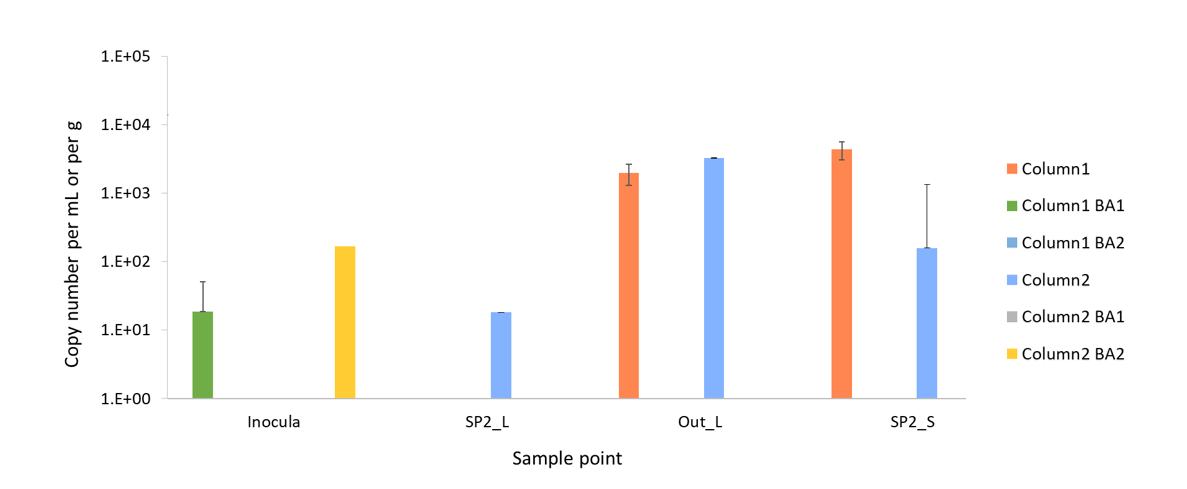


**Figure S2. Changes in *tfdA* concentration in the inoculum used for BA1 and BA2 inoculation and in the different sampling points from column1 and column2 before BA. Error bars represent the standard deviation among triplicate samples.**

| **A** |
| --- |
| **B** |

**Figure S3. Residual fluorescein concentration at different sampling points for A) Column 1 BA1; B) Column 2 BA1**

**References**

Bælum, J., Nicolaisen, M.H., Holben, W.E., Strobel, B.W., Sørensen, J., Jacobsen, C.S., 2008. Direct analysis of tfdA gene expression by indigenous bacteria in phenoxy acid amended agricultural soil. ISME J. 2, 677–687. https://doi.org/10.1038/ismej.2008.21
